# A Tale as Old as Time: Contraception Concerns in the 21st Century

**DOI:** 10.2147/OAJC.S581526

**Published:** 2026-04-07

**Authors:** Rachel Margaret Bottomley-Wise, Amie Woodward, Adenike Okanlawon, Amanda J Mason-Jones

**Affiliations:** 1 Department of Health Sciences, University of York, York, UK; 2 Institute for Health and Care Improvement, York St John University, York, UK

**Keywords:** mHealth, contraception, wearable device, ethics, reproductive justice

## Abstract

Historical inconsistencies in contraception research have led to concerns about side effects regarding hormonal contraception. Today, concerns about the hormonal contraceptive pill are still ongoing. The side effects from hormonal contraception and prevailing socioeconomic barriers to effective contraception have heralded in the use of Mobile Health (mHealth) applications as birth control methods. These applications are set to become ubiquitous with the proliferation of wearable devices that monitor biodata. Access to modern contraceptives depends on who you are and where you live and often has cost-implications. The use of “mHealth” applications has been rising in recent years and are marketed as having maximal reward for minimal cost and perhaps could alleviate some of these concerns. While mHealth applications are flexible, accessible, and low cost, this paper suggests that we exercise caution so as not to repeat past mistakes. We provide a critical review of the history of research into contraception. We may have come far, but we still have a long way to go for equitable access to contraceptive healthcare.

## Plain Language Summary

We provide an in-depth commentary on the history of contraceptive research. We summarise a journey from the early experimental studies on the hormonal contraceptive pill to the present situation and current challenges. We suggest where we hope contraception research will progress in the future, with a word of caution to not repeat past mistakes. We discuss more broadly how we need to uphold women’s rights to have control over their own bodies and their decision whether or not to have children. The rise of mobile health applications (apps) may help with some of the difficulties in accessing traditional contraceptives (eg, the associated costs and environmental concerns). The implications for mobile health application use for Low- and Middle-Income Countries have the potential to be very positive. However, if they are not carefully reviewed before approval, it often ends up being the most disadvantaged people that suffer from using apps that are not rigorously tested or may not be fit for purpose. We may seem to have come a long way, but we may not yet be at the stage where women are making fully informed contraceptive choices.

## Video abstract

**Figure:**
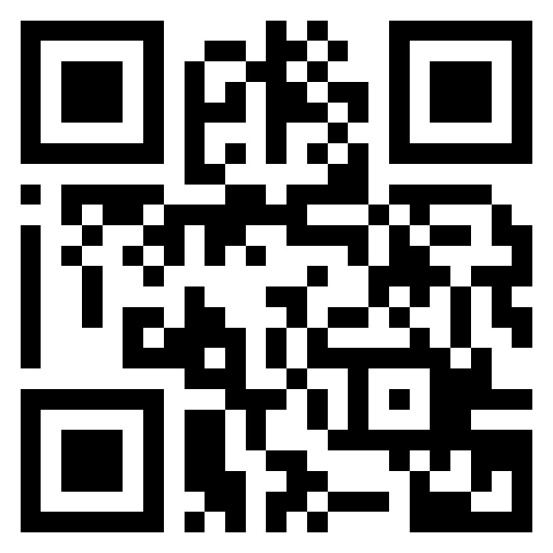

Point your SmartPhone at the code above. If you have a QR code reader, the video abstract will appear. Or use: https://youtu.be/BI6no9aCxHE

## Introduction

It is important to review the history of contraception research as its implications are still felt today. The history of contraceptive innovation has raised some ethical and biopolitical concerns which has been a source of critical and ongoing challenges that necessitate robust research processes.^1^ This commentary critically analyses the history and present state of contraception through the lens of Reproductive Justice.^2^ “Reproductive justice” refers to women having fair, equal opportunity, and control over their bodies, whether to have children or not, and to be able to raise children in a safe environment if they choose to have them.^3^ We examine the trajectory from genuine innovation to ethical breaches and the ongoing lack of access across populations, which reveals non-transparent research pathways to innovation development and approval, all of which fundamentally contradict the goal of reproductive autonomy. An approach informed by reproductive justice should therefore take primacy.^4^ Reproductive justice encompasses broader social, economic, and political contexts, beyond individual autonomy. It advocates for equitable access for all to reproductive healthcare, breaking down systemic barriers, and recognising the unique experiences of individuals. By centring on reproductive justice, an inclusive and empowering framework for decision-making can be created in this critical domain.^2^ We also look towards the future of contraception in this digital age, and their associated challenges such as, data privacy, regulatory issues, and conflicts of interest. We conclude that only by reflecting on the past and applying a critical lens to present inequities can we ensure that future contraceptive technologies are developed through robust, transparent, and user-centred processes, guaranteeing truly equitable healthcare.

### Reproductive Rights and Contraception

Sexual and reproductive health rights enshrine access to health services, education and autonomy.^5^ However, additional commitment worldwide is needed to meet the United Nations Sustainable Development target which focusses on integrating universal access to sexual and reproductive health care services into national strategies and programmes.^6–8^ The “Women’s Health Strategy for England”, for example, has set out a 10-year action plan to improve the health and wellbeing of women and girls across all stages of life,^9^ with contraception highlighted as a crucial component at all three life stages (adolescence, middle years, and later years).^9^ The “State of World Population Report”^10^ suggests that contraception is the most obvious way to prevent unintended pregnancy. Health care providers have an important role in ensuring access to high-quality reproductive health care services free from judgement,^11^ although, barriers remain for many people.^12^,^13^ There can be a conflict between people’s embodied knowledge about their reproductive lives and healthcare providers' knowledge and attitudes.^14^ Although people may be purported to be at the centre of medical care, unequal power dynamics remain.^14^ This means that autonomy in reproductive decision-making may be hampered.^15^ It is not surprising that some women feel a lack of trust regarding the medical profession and side effects of contraceptives, when we look at the history of contraceptives. As one such example, “The Dalkon Shield” intrauterine device (IUD), introduced in 1971, was terminated after three years as it resulted in pelvic inflammatory disease, permanent infertility and the deaths of users.^16^,^17^ Nevertheless, when the safety of the Dalkon Shield was questioned,^18^ and its use stopped in the US, it continued to be prescribed in over forty other countries.^17^

Users of contraception often have concerns about side effects and draw on embodied knowledge to guide medical decision-making about their bodies.^15^,^19^ We discuss this in more detail in the next section, where we use the example of the oral contraceptive pill to clearly illustrate how risks and benefits need to be clearly communicated to women and tested in empirical studies so they can make informed contraceptive choices.

### The Oral Contraceptive Pill – Concerns Over Side Effects

It is difficult to find a clear consensus on the risks and benefits of the oral contraceptive pill, which is a hormonal, temporary method of contraception. For a list of the different types of contraception and whether they are hormonal or non-hormonal, please refer to Supplementary Materials 1.^20^ For every article that discusses side effects of the pill, there are numerous articles discussing the benefits of the contraceptive pill and often this research is contradictory. For example, around the time of the early experimental studies of the contraceptive pill which showed some side effects, scientists were accused of falsifying a “pill scare” and concerns raised about the oral contraceptive pill were rigorously defended by pharmaceutical companies involved at that time.^21^ As described in Vandenbroucke et al’s^21^ commentary, at the end of 1998 three major studies conducted without sponsorship, from the contraceptive pill producing pharmaceutical industry, found a higher risk of venous thromboembolism (VTE) for third generation contraceptives, but this was not found with three sponsored studies.^22^ In 1995, four studies found the same risk.^21^ The companies claimed that the “pill scare” was potentially a repercussion of providing inadequate information and misinformation from the media, as the risk of VTE from combined oral contraceptives was not understood in context.^23^ Many women may then think a well-known side effect of the pill is VTE. However, the risk of VTE in a normal pregnancy is approximately 30 in 10,000, with the use of the oral contraceptive pill it is 9–10 in 10,000.^24^ Taking the pill lowers a woman’s risk of VTE compared to getting pregnant, yet, this protective aspect of taking the pill is rarely discussed.^1^ This highlights the necessity for robust processes in research that provide users of products with enough accessible information to make informed choices about the products they use for their reproductive health. Nevertheless, scientific concerns raised decades ago still remain. For instance, a recent nested case-control study provided new evidence that use of progestogen-only contraceptives was associated with breast cancer risk similar to the combined hormonal contraceptives.^25^ Prolonged use of the oral contraceptive pill has also been associated with the risk of cervical cancer.^26^ However, a systematic review found a 50% decrease in risk of endometrial cancer from taking combined oral contraceptives.^27^ For most of the studies in their review, this protective effect persisted for between 10 and 20 years after ceasing to use the combined oral contraceptives.^27^ The risks and benefits of the oral contraceptive pill are nuanced depending on pre-existing conditions and reporting and interpretation of studies is often inconsistent. This highlights the need for further empirical research such as systematic reviews and randomised controlled trials on the risks and benefits and for these to be clearly communicated to women so they can make informed contraceptive choices.

Although it is accepted that conclusions from research may shift over time with greater evidence, types of controversy such as the “pill scare” can affect public perception and policy for decades to come. For example, public concerns about the safety of hormonal contraception and developing side-effects, particularly menstrual related side effects, may cause adolescents and young people to feel that not only is their general health being affected but their reproductive health too.^28^ Examples of cited inaccuracies expressed in a survey about the contraceptive pill include “It can mess up the girl’s insides”.^28^ However, a systematic review of the depot medroxyprogesterone acetate (DMPA, a hormonal, temporary contraception), often known by the brand name Depo-Provera i.e. the hormonal injection, and the subdermal levonorgestrel implant, found normal menstruation patterns were experienced by only 23% of women using the implant and 11% of women using the hormonal injection.^29^ Thus, there does appear to be some basis for women’s fears that certain hormonal contraceptives will affect their periods. Bleeding changes with hormonal contraceptives are common and unpredictable, especially for those using DMPA injections, hormonal implants, hormonal intrauterine devices (IUDs), and the progestogen-only pill.^23^ Women should be informed that bleeding may not resolve within the first six months, and alternative treatments options should be discussed.^23^ However, combined hormonal contraceptive pills can regulate the menstrual cycle, reduce bleeding and pain from menstruation, and have other benefits such as helping with acne, and protecting against ovarian and endometrial cancer.^23^ Recently, the DMPA injection has been in the press as some users have developed much higher relative risk of meningiomas (a type of typically benign tumour), but the overall risk is still low.^30^ A number of key misconceptions surround hormonal contraception focusing on issues such as mental health, sexuality, infertility, safety and destigmatisation of side effects, for some of these themes there is evidence justifying them, but in many cases the benefits of the contraception may outweigh the risks.^23^

## The Present–Current Challenges in Contraception

Today, it may seem that contraception is easily accessible, free, and has fewer side effects than before. However, there are still prevailing socioeconomic barriers to effective contraception.

### Contraceptive “Choice”

Despite women needing and wanting contraception, many women do not have access to modern contraception methods^31^ or the ability to control their fertility.^32^ How readily available and affordable modern contraception is depends on where you live and who you are. This is particularly pronounced for vulnerable populations, such as those in incarceration, who encounter additional challenges beyond financial constraints, including limited freedom and restricted access.^33^ A survey that explored changes in methods used after injectable contraception was introduced in the early 1990s, found the prevalence rates for injectables and other modern contraception methods varied widely over time and by country.^34^ For example, over the study period, modern contraceptive method use increased by just 5% in Kenya, but by as much as 27% in Namibia. However, differences in the freedom of choice women have often had financial implications.^34^ Although it is said that inexpensive, permanent, long-acting methods adopted at younger ages result in lower costs than injectable or other expensive, short-acting methods^34^ ready access to appropriate health systems and services means that reproductive freedom remains a distant goal for many. Policies on IUDs differ such that for those having to pay privately they often remain out of reach.^35^ The present financial barriers to access are one of the more challenging aspects to navigate as financial interventions prove to be effective in closing the access gap. A study conducted in Burkina Faso^36^ highlighted that removing user fees was a highly effective strategy for significantly enhancing the population’s access to family planning services especially in Low- and Middle-Income Countries (LMICs). However, the current lack of uniform policies and public awareness of policies (if any) exacerbates the challenges faced by those seeking accessible – and affordable – contraceptive options.^37^

## The “Future” of Contraception

### Innovation and the Rise of the Mobile Health Application

One potential way to help overcome some of the barriers to accessing contraception may be the use of mobile health applications. The use of mobile technology to provide health care or “mHealth apps” has been steadily increasing in recent years and are marketed as having maximal reward for minimal cost.^38^ Users often choose them because they are low cost and because of environmental concerns.^39^ For instance, some “traditional” contraceptives such as most condoms, are non-biodegradable. Innovators have developed “mHealth apps” as contraceptive tools, capitalising on the widespread adoption of smartphones, especially among adolescents and young people who are deemed to be at a higher risk of unintended pregnancy and have high engagement with smart devices.^40^ The apps serve various purposes, including fertility tracking, birth control reminders, provision of sexual and reproductive health (SRH) information, and some offer details on contraceptives and related services.^40^,^41^

Contraceptive and family planning apps serve to provide more people with access to their preferred method of contraception.^42^ This is particularly beneficial in LMICs where preventable maternal death from complications in pregnancy and childbirth occur at a higher rate.^43^ Key reasons for unmet family planning needs are a lack of knowledge of and access to modern contraceptive methods.^43^ However, the use of smartphones has proliferated, including in LMICs, and family planning apps bridge this gap and deliver both information and access.^44^

Despite these potential benefits, a recent review of family planning mHealth interventions found little evidence of long-term contraceptive behaviour change even though the apps improved participant knowledge and attitudes to modern contraceptive methods.^42^ These results are replicated in a review of mHealth intervention and contraceptive behaviour change in LMICs; fewer than half of the studies included found improvements in family planning outcomes.^43^ These results suggest that there are implementation problems and/or that purported behaviour change theories or techniques embedded into the apps have not been properly tested against the behaviour they aim to change.

In 2023, a well-known technology company partnered with a well-known contraception app to bring fertility tracking to its wearable devices, making the app technology available in 32 countries worldwide.^45^ This year, this well-known technology company is poised to release its long awaited smart ring, which is slated to continue the partnership between this company and this app.^46^ The app in question is a popular, and somewhat controversial,^47^ digital fertility tracking application that was first available for iPhone Operating System (iOS) and Android mobile devices. It is regarded as the first Food and Drug Administration (FDA) approved app-based contraceptive with a CE mark.^47^ The app aims to assist users with fertility awareness-based methods of family planning and works by users inputting details about their menstruation and daily basal body temperature. The algorithm then uses this information to predict ovulation.

The use of in-built temperature tracking technology paired with the fertility tracking algorithm from the app offers an unprecedented opportunity to provide accessible, non-hormonal contraception and family planning, and removes an element of user error in taking temperature automatically.

Indeed, when this well-known contraception app was approved by the FDA, a question arose about the robustness of the FDA approval standards as they had allowed a less rigorous, more “streamlined” process for the approval of contraceptive devices.^48^ They reviewed this app through the “de nova premarket review pathway” for novel, low-to-moderate-risk devices of a new type.^49^ Meanwhile, the National Institute for Health and Care Excellence (NICE) in the UK provided a ‘Medtech innovation briefing’^47^ on the app in question. This report highlighted that three out of four clinical academic specialists consulted suggested that there was a need for further evidence before the digital application could be adopted by the National Health Service (NHS).^47^ Reports from those using the app have been mixed, with those who experienced unplanned pregnancies reporting feelings of shame, naivety, and feeling like they had been targeted as a consumer rather than a person seeking healthcare.^50^ The app’s website lists research by medical experts but much of this research has been conducted by people who own stock, are employed by the company^51–53^ or are affiliated with the company.^54^ However, as this well-known contraception app has not been tested in a randomised controlled trial against other contraceptive methods, this calls into question how substantial the evidence is supporting the effectiveness claims of the company^48^ and precludes the use of the company’s trial data from meta-analyses on contraceptive effectiveness.^55^ Similar to the introduction of the “Dalkon Shield” in 1971, the introduction of mHealth apps highlight the necessity for robust, transparent research that provides women with the tools to make informed choices about their contraception practices. We may have come far, but we still have a long way to go for equitable healthcare.

Earle et al^56^ conducted a scoping review of menstruation and fertility app trackers. One app mentioned in their review has been evaluated in a study by Haile et al^57^ which is based on the Standard Days Method, using first date of menstruation only. Their study draws on data from LMICs and highlights that the use of the app was significantly influenced by country and age of the user. This mirrors our findings from other modern contraceptives, such as the IUD, that access to contraception varies depending on who you are and where you come from. Earle et al^56^ described how the app can be distributed at low cost and may expand access to fertility-based awareness methods. The majority of app users were between 20 and 29 years old, 39.9% were using the app to prevent pregnancy, 38.5% to plan a pregnancy, and 21.6% to track their cycle.

Another app in their review estimates fertile days only using date of menstruation. The app uses Dynamic Optimal Timing (DOT) to pinpoint the days with the highest likelihood of becoming pregnant. Li et al^58^ discuss several simulation studies using this app to prevent pregnancy. The app has not received FDA approval or European certification, and there are concerns about using the app to prevent pregnancies.^59^

### The Number of Red “Danger” Days

Mobile apps like the ones mentioned differ in terms of the underlying method they use to calculate fertile days, for instance, the Standard Days Method, DOT, or the Rhythm Method could be used. According to information published on their website, one popular contraceptive app, using their specific algorithm, reduced the likelihood of receiving a wrong non-fertile “red” day on the most fertile days by 69% compared to the Rhythm Method.^60^ This app’s algorithm differs from the Rhythm Method as it uses basal body temperate data and learns from the pattern of the user’s cycle to predict the fertile window.^60^ Compared to the Standard Days Method, the likelihood of receiving a wrong non-fertile “red” day reduced by 97% when using this popular app instead of standard days.^60^ The number of non-fertile “red” days varies between the methods too. This popular app and the Standard Days Method both give a similar number of red days (56% and 58% on average) while the Rhythm Method only provided 17% “red” days during the first year of use.^60^ This calls into question the accuracy of some apps for instance the app evaluated in the study by Haile et al^57^ that was based on the Standard Days Method, using first date of menstruation only.

### Ongoing Challenges with Contraceptive Apps

As mentioned, mHealth apps may have the potential to help overcome some of the barriers to accessing contraception, as they are marketed as having maximal reward for minimal cost.^38^ Until recently, however, the only way for developing countries to access the internet would have been through expensive smart phones,^61^ designed in and for developed countries, you generally need an active internet connection to download and install apps on Android and iOS Devices. Presently, the smart feature phone with internet connectivity has been specifically designed with those with low income in developing countries in mind.^61^ For example, the JioPhone has become available to millions of people in India who have low incomes.^61^ However, the increase in “Femtech” in India has failed to recognise the structural inequalities and socio-economic disadvantages that exist when accessing healthcare.^62^ Poorly defined data privacy policies in India allow for users’ bodies to become commercialised as Femtech tries to maximise commercial gain and personal data can also be breached, which has repercussions for autonomy and the dignity of its users.^62^

There is a need for independent research that is free from commercial interest and risk.^56^ Women also may be using apps to prevent pregnancy when they have not been designed for this purpose, for instance most fertility apps are not underpinned by evidence-based fertility-based awareness methods and often include a disclaimer discouraging use for avoiding a pregnancy.^63^ Fertility services remain out of reach for many individuals due to social barriers, eligibility, stigma and cost, so digital fertility management platforms may be a promising disruption to the existing industry,^64^ and place the power back onto women to manage their own fertility. Yet, some of the language in these apps encourages women not to trust their own bodies and compares the intelligence of these digital tracking apps to specialist medical professionals.^64^ There are also concerns about harvesting and selling of user data, and paid subscription services.^64^

Further, privacy and security vulnerabilities in fertility-monitoring apps and menstrual cycle tracking are significant challenges and may present risks such as accidental pregnancy or legal consequences.^65^ These risks highlight the need for increased transparency and accountability of digital apps for women’s health.^65^ Like with the early studies on contraception, the need for transparency and accountability in digital apps is fundamental to helping women make informed choices about their contraceptive methods.

Contraceptive apps enable women to track multiple symptoms eg, the date of menstruation, cervical mucus, basal body temperature; however, this plethora of symptoms can cause users to experience “tracker fatigue”.^56^ This could negatively influence the effectiveness of the apps as the data needs to be inputted consistently and to a high degree of accuracy.^56^ Further, women may turn to such apps to track their cycles for a variety of reasons, and these reasons may change over time.^56^ For instance, they may wish to track their cycle for medical reasons, to understand their body and learn about their cycle, rather than just for contraceptive purposes.

Unlike other modern contraceptives (eg, the implant, injection, or IUD) contraceptive apps relying on fertility awareness-based methods require diligence and daily tracking (eg, temperature, cervical mucus, length of period, etc). This calls into question whether the “perfect use” estimate of the apps’ effectiveness in preventing pregnancy differs from the “typical use” estimates. In one popular contraceptive app, the perfect-use failure rate was calculated as 1.0% whereas the typical-use failure rate was calculated as 5.8%.^66^ However, the app only requires a user to enter her menstrual period start date, which is less burdensome than other fertility awareness-based methods.^66^ Another popular contraceptive app had a “typical use” pearl index of 6.9 pregnancies per 100 woman-years compared to 1.0 for the “perfect use” estimate.^54^ When compared to the IUD, which once inserted requires no further action on the part of the individual, the effectiveness of both the hormonal and non-hormonal IUD at preventing pregnancy is more than 99%.^67^ NICE provides efficacy rates of the available contraceptive methods, and the Levonorgestrel (hormonal) IUD has a “typical use” and “perfect use” efficacy rating of 0.2% for the percentage of women experiencing an unintended pregnancy within the first year of use, this is 0.8% “typical use” and 0.6% “perfect use” for the copper IUD respectively.^68^ Fertility awareness methods, in general, have a “typical use” estimate of 24% and a “perfect use” estimate of 1–9%.^68^ This need to perfectly use the app for it be as effective as other contraceptives may affect its popularity and people may discontinue using them due to feeling the daily burden of inputting multiple types of data. For instance, over a 12-month period, 54% of people discontinued using this popular contraceptive app.^54^ The discontinuation rate per month was constant over the year, except for cycle 0 when very few women discontinued.^54^

In another study, 41.9% of users were still using the app in question at the end of their follow-up period, contributing on average 13 menstrual cycles of data.^52^ As well as looking at the number of people who discontinue using contraceptive apps, it is interesting to explore the characteristics of people who turn to these apps as their primary method of contraception. It could be argued that as medical abortion in some developed parts of the world is easier to access than in the past, women are happier to use a riskier contraceptive method if it means they can avoid the side effects associated with hormonal methods. However, when exploring 12,247 women in the UK using this well-known contraceptive app, they have a mean age of 30.3, mean BMI was in the healthy range of 23.4, 83.2% report being in a relationship, 83% are educated to university degree level or higher, 84.5% did not currently have children.^52^ When exploring methods of contraception on fertile days, 58.3% reported using condoms, 9.3% abstain from sex, and 23.3% use withdrawal which is typically a low effective method to prevent a pregnancy.^52^ Another cohort study including 5879 women in the US who contributed an average of 10.5 months of data found similar results. The average user of this well-known contraceptive app was 30 years old, with a BMI in the healthy range, reported being in a stable relationship, and was educated to university degree level.^51^ This same study found on fertile days, 52.8% used a condom, 10.9% abstain, and 25.2% used the withdrawal method. The high use of withdrawal, both papers suggest, indicates the potential of needing more sexual education for users of this app. Contraceptive apps could provide a platform for education around fertility, women’s health, and contraception.^52^ Rather than these women risking unwanted pregnancy, it could be that as the women were educated, in stable relationships, in their 30s, they may have potentially turned to this app, with the notion that if they did become pregnant they were in a stable situation to continue with the pregnancy. Perhaps, the option to learn more about their cycle, or to be able to switch the app to try to conceive rather than as contraception at a later date, may appeal to some users. For example, the website for this popular app claims that the same science can be used to plan a pregnancy when the time is right,^67^ which may be a welcome addition for some users. This warrants further qualitative research.

### Coercion and Sex

As well as making informed choices on the contraception methods, women also should be able to choose when to have and when to abstain from sex. Fertility awareness-based methods require individuals to abstain from sex on “red” days, when they would be most likely to get pregnant. This requires a certain degree of cooperation between both partners, to mutually agree to not have sex on the specified days. There is evidence to suggest that men may sometimes use coercion despite clear signs of refusal for sex from their partners, and in these instances, they may still believe the sexual act to be consensual.^69^ Although women can say no to sex, this overlooks the notion that men can use coercion even though a refusal has been given.^69^ An analysis of in-depth semi structured interviews with seven male Australians found that men understood refusals for sex but were naive regarding their use of coercion, or that they employed language that minimised or justified their use of coercion and the impacts that this may have.^69^ If contraceptive apps using fertility awareness-based methods increase in popularity in the future, then education for both members of the couple around coercion would be warranted. Perhaps, these applications could include resources and educational videos on avoiding coercion and understanding consent.

## Conclusions

Universal access to safe and voluntary family planning methods is the best way to promote gender equality, autonomy for women, and reduce poverty. When reflecting on the oral contraceptive pill, we can see a need to provide information about risks and side-effects. Concerns about the hormonal contraceptive pill are still ongoing and reproductive justice has not yet been achieved for all. Access to modern contraceptives still depends on who you are and where you live. To help bridge the gap, mHealth apps and fertility tracking technology are set to become increasingly prevalent with the increasing popularity in wearable devices (watches, rings) that read and monitor biodata. This provides an excellent opportunity for widely accessible, non-hormonal contraceptive and family planning autonomy that bypasses traditional barriers within healthcare systems. Although individuals should anticipate crucial developments in reproductive health technology, the concept of ‘Techno-utopia’^70^ in reproductive health often over-promises and underdelivers,^71^ which may disadvantage users who have limited resources or understanding. The voices of those affected by this, women, need to be reflected highly in all stages of this research to improve the overall quality and acceptability of the research.^72^ This approach is crucial as it ensures women’s active involvement in the decision-making process regarding interventions to improve their reproductive health and knowledge thereof, as well as overall quality of life. We may seem to have come a long way, but women are still being misled by bold advertising claims made without sufficient evidence and dubious marketing practices.

## Data Availability

No new data were generated or analysed in support of this research.

## Abbreviations

Apps, Application; FDA, Food and Drug Administration; DMPA, Depot Medroxyprogesterone Acetate; DOT, Dynamic Optimal Timing; IUD, Intrauterine Device; IUDs, Intrauterine Devices; iOS, iPhone Operating System; LMICs, Low- and Middle-Income Countries; mHealth, Mobile Health; NHS, National Health Service; NICE, National Institute for Health and Care Excellence; SRH, Sexual and Reproductive Health; VTE, Venous Thromboembolism.
